# (2*E*)-2-[(3*E*)-4-Phenyl­but-3-en-2-yl­idene]hydrazinecarboxamide

**DOI:** 10.1107/S160053681105255X

**Published:** 2011-12-10

**Authors:** S. Samshuddin, Ray J. Butcher, Sema Ozturk Yıldırım, Mehmet Akkurt, B. Narayana, H. S. Yathirajan

**Affiliations:** a Department of Studies in Chemistry, Mangalore University, Mangalagangotri, Mangalore 574 199, India; b Department of Chemistry, Howard University, 525 College Street NW, Washington, DC 20059, USA; c Department of Chemistry, Howard University, 525 College Street NW, Washington, DC 20059, USA, and, Department of Physics, Faculty of Sciences, Erciyes University, 38039 Kayseri, Turkey; d Department of Physics, Faculty of Sciences, Erciyes University, 38039 Kayseri, Turkey; e Department of Studies in Chemistry, University of Mysore, Manasagangotri, Mysore 570 006, India

## Abstract

In the title compound, C_11_H_13_N_3_O, the phenyl ring is disordered over two sites, with occupancy factors in a 0.520 (17):0.480 (17) ratio. The dihedral angle between the ring planes of the major and minor components of the disordered ring is 12.9 (2)°. In the crystal, mol­ecules are linked by N—H⋯O hydrogen bonds, forming *R*_2_^2^(8) ring motifs. C—H⋯O, C—H⋯N and C—H⋯π inter­actions also occur.

## Related literature

For background to the biological activity of semicarbazones, see: Beraldo *et al.* (2002 ▶); Teixeira *et al.* (2003 ▶); Du *et al.* (2004 ▶); Kucukguzel *et al.* (2006 ▶); Beraldo & Gambino (2004 ▶). For related structures, see: Naik & Palenik (1974 ▶); Wang *et al.* (2004 ▶); Yathirajan *et al.* (2006 ▶); Sarojini *et al.* (2007 ▶).
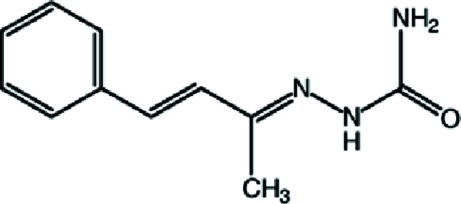


## Experimental

### 

#### Crystal data

C_11_H_13_N_3_O
*M* *_r_* = 203.24
Monoclinic, 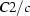

*a* = 15.1094 (8) Å
*b* = 24.4445 (11) Å
*c* = 7.0368 (4) Å
β = 109.908 (6)°
*V* = 2443.7 (2) Å^3^
*Z* = 8
Mo *K*α radiation
μ = 0.07 mm^−1^
*T* = 123 K
0.40 × 0.30 × 0.18 mm

#### Data collection

Oxford Diffraction Xcalibur Ruby Gemini diffractometer
Absorption correction: multi-scan (*CrysAlis RED*; Oxford Diffraction, 2007 ▶) *T*_min_ = 0.987, *T*_max_ = 1.000
12712 measured reflections
3528 independent reflections
2748 reflections with *I* > 2σ(*I*)
*R*_int_ = 0.026

#### Refinement

*R*[*F* ^2^ > 2σ(*F* ^2^)] = 0.055
*wR*(*F* ^2^) = 0.176
*S* = 1.05
3528 reflections
168 parameters
H-atom parameters constrained
Δρ_max_ = 0.26 e Å^−3^
Δρ_min_ = −0.22 e Å^−3^

### 

Data collection: *CrysAlis PRO* (Oxford Diffraction, 2007 ▶); cell refinement: *CrysAlis PRO*; data reduction: *CrysAlis RED* (Oxford Diffraction, 2007 ▶); program(s) used to solve structure: *SHELXS97* (Sheldrick, 2008 ▶); program(s) used to refine structure: *SHELXL97* (Sheldrick, 2008 ▶); molecular graphics: *ORTEP-3* (Farrugia, 1997 ▶); software used to prepare material for publication: *WinGX* (Farrugia, 1999 ▶) and *PLATON* (Spek, 2009 ▶).

## Supplementary Material

Crystal structure: contains datablock(s) global, I. DOI: 10.1107/S160053681105255X/tk5032sup1.cif

Structure factors: contains datablock(s) I. DOI: 10.1107/S160053681105255X/tk5032Isup2.hkl

Supplementary material file. DOI: 10.1107/S160053681105255X/tk5032Isup3.cml

Additional supplementary materials:  crystallographic information; 3D view; checkCIF report

## Figures and Tables

**Table 1:** Hydrogen-bond geometry (Å, °) *Cg*1 and *Cg*2 are the centroids of the disordered benzene rings C1*A* –C6*A* and C1*B*–C6*B*, respectively.

*D*—H⋯*A*	*D*—H	H⋯*A*	*D*⋯*A*	*D*—H⋯*A*
N2—H2*B*⋯O1^i^	0.88	2.12	2.9785 (15)	166
N3—H3*B*⋯O1^ii^	0.88	2.08	2.9434 (14)	168
C10—H10*A*⋯O1^i^	0.98	2.51	3.2384 (17)	131
C10—H10*B*⋯N1^iii^	0.98	2.58	3.4566 (19)	148
C4*B*—H4*BA*⋯*Cg*1^iv^	0.95	2.86	3.618 (5)	138
C4*A*—H4*AA*⋯*Cg*1^iv^	0.95	2.76	3.590 (5)	146
C4*A*—H4*AA*⋯*Cg*2^iv^	0.95	2.93	3.714 (5)	141

Symmetry codes: (i) 
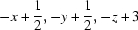
; (ii) 
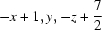
; (iii) 
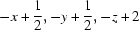
; (iv) 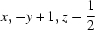.

**Fig. 1.**
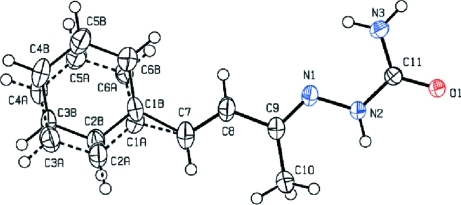
The disordered molecule (I) showing the atom labeling scheme. Atoms of the minor disorder components are joined with dashed lines. Displacement ellipsoids for non-H atoms are drawn at the 50% probability level.

**Fig. 2.**
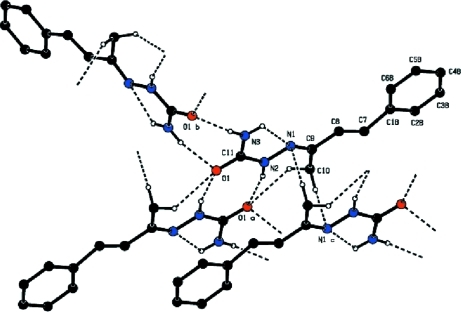
View of the N—H···O mediated dimers in (I) and their connections to other molecules by C—H···O and C—H···O hydrogen bonding.

## supplementary crystallographic information

### Comment

Semicarbazones presents a wide range of biological applications such as antitumoral, anticonvulsant, anti-trypanosomal, herbicidal and biocidal activities (Beraldo & Gambino, 2004; Beraldo *et al.*, 2002; Teixeira *et al.*, 2003). They can also be used as important intermediates in organic synthesis, mainly for obtaining heterocycle rings, such as thiazolidones, oxadiazoles, pyrazolidones, and thiadiazoles (Du *et al.*, 2004; Kucukguzel *et al.*, 2006)

Crystal structures of some semicarbazone derivatives, *viz*., acetone semicarbazone and benzaldehyde semicarbazone (Naik & Palenik, 1974); 3,4- methylenedioxybenzaldehyde semicarbazone (Wang *et al.*,2004); 4-(methylsulfanyl)benzaldehyde thiosemicarbazone (Yathirajan *et al.*, 2006) and 4-(Methylsulfanyl)benzaldehyde semicarbazone (Sarojini *et al.*, 2007) have been reported. In view of the importance of semicarbazones, the title compound (I) was prepared and its crystal structure is reported.

Fig. 1 shows the molecular structure of the title compound (I) with the disordered phenyl ring. The dihedral angle between the major and minor disorder components of the phenyl ring is 12.9 (2)°. The C7—C8—C9—C10, C7—C8—C9—N1, C10—C9—N1—N2, C8—C9—N1—N2, N1—N2—C11—N3 and N1—N2—C11—O1 torsion angles are -2.7 (2), 178.13 (13), -1.37 (19), 179.53 (10), -1.40 (17) and 179.22 (11)°, respectively, and indicate planarity in the molecule.

In the crystal, the molecules form centrosymmetric dimers with an *R*_2_^2^(8) ring motif through a pair of N—H···O hydrogen bonds. These dimers are further connected into a three-dimensional network by intermolecular C—H···O and C—H···N hydrogen bonds (Table 1, Fig. 2). Weak intermolecular C—H···π interactions further stabilize the crystal structure.

### Experimental

To a mixture of a benzylidene acetone (1.46 g, 0.01 mol) and semicarbazide hydrochloride (1.12 g, 0.01 mol) in 50 ml ethanol was added a sodium acetate solution (2 g in 5 ml water) which was then refluxed for 4 h. The resultant solution was concentrated to half of its volume and poured into 50 ml ice-cold water. The precipitate thus formed was collected by filtration and purified by recrystallization from ethanol. The single crystal was grown from its absolute alcohol solution by slow evaporation. The yield was 74%. (M.pt. 455–459 K).

### Refinement

The phenyl ring is disordered over two positions with refined site occupancies of 0.520 (17) and 0.480 (17). All H atoms were placed in idealised positions and refined in the riding model approximation [N—H = 0.88 Å, aromatic C—H = 0.95 Å and methyl C—H = 0.98 Å, and with *U*_iso_(H) = 1.2 or 1.5 *U*_eq_(parent atom)]. In the crystal structure, there is an 206 Å^3^ void, but the low electron density (0.26 e.Å^-3^) in the difference Fourier map suggests no solvent molecule occupying this void.

### Crystal data

**Table:**

C_11_H_13_N_3_O	*F*(000) = 864
*M**_r_* = 203.24	*D*_x_ = 1.105 Mg m^−^^3^
Monoclinic, *C*2/*c*	Mo *K*α radiation, λ = 0.71073 Å
Hall symbol: -C 2yc	Cell parameters from 5469 reflections
*a* = 15.1094 (8) Å	θ = 3.0–30.9°
*b* = 24.4445 (11) Å	µ = 0.07 mm^−^^1^
*c* = 7.0368 (4) Å	*T* = 123 K
β = 109.908 (6)°	Prism, colourless
*V* = 2443.7 (2) Å^3^	0.40 × 0.30 × 0.18 mm
*Z* = 8	

### Data collection

**Table:**

Oxford Diffraction Xcalibur Ruby Gemini diffractometer	3528 independent reflections
Radiation source: Enhance (Mo) X-ray Source	2748 reflections with *I* > 2σ(*I*)
graphite	*R*_int_ = 0.026
Detector resolution: 10.5081 pixels mm^-1^	θ_max_ = 30.9°, θ_min_ = 3.0°
ω scans	*h* = −20→20
Absorption correction: multi-scan (*CrysAlis RED*; Oxford Diffraction, 2007)	*k* = −34→26
*T*_min_ = 0.987, *T*_max_ = 1.000	*l* = −7→9
12712 measured reflections	

### Refinement

**Table:**

Refinement on *F*^2^	Primary atom site location: structure-invariant direct methods
Least-squares matrix: full	Secondary atom site location: difference Fourier map
*R*[*F*^2^ > 2σ(*F*^2^)] = 0.055	Hydrogen site location: inferred from neighbouring sites
*wR*(*F*^2^) = 0.176	H-atom parameters constrained
*S* = 1.05	*w* = 1/[σ^2^(*F*_o_^2^) + (0.0981*P*)^2^ + 0.6768*P*] where *P* = (*F*_o_^2^ + 2*F*_c_^2^)/3
3528 reflections	(Δ/σ)_max_ < 0.001
168 parameters	Δρ_max_ = 0.26 e Å^−^^3^
0 restraints	Δρ_min_ = −0.22 e Å^−^^3^

### Special details

**Table:**

Geometry. Bond distances, angles *etc*. have been calculated using the rounded fractional coordinates. All su's are estimated from the variances of the (full) variance-covariance matrix. The cell e.s.d.'s are taken into account in the estimation of distances, angles and torsion angles
Refinement. Refinement on *F*^2^ for ALL reflections except those flagged by the user for potential systematic errors. Weighted *R*-factors *wR* and all goodnesses of fit *S* are based on *F*^2^, conventional *R*-factors *R* are based on *F*, with *F* set to zero for negative *F*^2^. The observed criterion of *F*^2^ > σ(*F*^2^) is used only for calculating -*R*-factor-obs *etc*. and is not relevant to the choice of reflections for refinement. *R*-factors based on *F*^2^ are statistically about twice as large as those based on *F*, and *R*-factors based on ALL data will be even larger.

### Fractional atomic coordinates and isotropic or equivalent isotropic displacement parameters (Å^2^)

**Table:**

	*x*	*y*	*z*	*U*_iso_*/*U*_eq_	Occ. (<1)
O1	0.36888 (6)	0.23438 (4)	1.62948 (12)	0.0323 (3)	
N1	0.34428 (7)	0.30220 (5)	1.17621 (15)	0.0337 (3)	
N2	0.31934 (7)	0.27704 (4)	1.32596 (14)	0.0310 (3)	
N3	0.47771 (7)	0.26174 (6)	1.49376 (16)	0.0458 (4)	
C1B	0.3063 (5)	0.40506 (19)	0.5724 (7)	0.0301 (9)	0.520 (17)
C2B	0.2428 (5)	0.4316 (2)	0.4074 (6)	0.0354 (10)	0.520 (17)
C3B	0.2747 (6)	0.45824 (19)	0.2685 (6)	0.0405 (13)	0.520 (17)
C4B	0.3701 (6)	0.45829 (14)	0.2947 (8)	0.0409 (13)	0.520 (17)
C5B	0.4336 (6)	0.4317 (2)	0.4598 (11)	0.0469 (14)	0.520 (17)
C6B	0.4017 (5)	0.4051 (2)	0.5986 (11)	0.0434 (11)	0.520 (17)
C7	0.25908 (11)	0.37670 (5)	0.71144 (19)	0.0393 (4)	
C8	0.31231 (10)	0.35103 (5)	0.87866 (19)	0.0375 (4)	
C9	0.27918 (9)	0.32596 (5)	1.03065 (18)	0.0327 (3)	
C10	0.17796 (10)	0.32953 (6)	1.0129 (2)	0.0395 (4)	
C11	0.38883 (8)	0.25686 (5)	1.49014 (17)	0.0304 (3)	
C3A	0.2443 (5)	0.4630 (2)	0.2639 (7)	0.0458 (13)	0.480 (17)
C4A	0.3345 (6)	0.45553 (15)	0.2576 (7)	0.0378 (13)	0.480 (17)
C5A	0.3972 (6)	0.4208 (2)	0.3956 (10)	0.0408 (14)	0.480 (17)
C6A	0.3697 (5)	0.39363 (19)	0.5398 (9)	0.0337 (11)	0.480 (17)
C2A	0.2169 (4)	0.4358 (2)	0.4081 (7)	0.0402 (11)	0.480 (17)
C1A	0.2795 (4)	0.40110 (19)	0.5461 (6)	0.0276 (10)	0.480 (17)
H7A	0.19470	0.37930	0.69980	0.0470*	
H3BA	0.23130	0.47640	0.15570	0.0490*	0.520 (17)
H5BA	0.49890	0.43180	0.47770	0.0560*	0.520 (17)
H6BA	0.44510	0.38700	0.71150	0.0520*	0.520 (17)
H10A	0.17330	0.33360	1.14770	0.0590*	
H10B	0.14520	0.29610	0.94940	0.0590*	
H10C	0.14890	0.36120	0.92980	0.0590*	
H4BA	0.39200	0.47650	0.19980	0.0490*	0.520 (17)
H8A	0.37790	0.34890	0.90100	0.0450*	
H2B	0.25980	0.27410	1.31570	0.0370*	
H3B	0.52440	0.24890	1.59690	0.0550*	
H3C	0.48940	0.27780	1.39300	0.0550*	
H2BA	0.17760	0.43160	0.38950	0.0420*	0.520 (17)
H2AA	0.15520	0.44090	0.41240	0.0480*	0.480 (17)
H3AA	0.20150	0.48670	0.16960	0.0550*	0.480 (17)
H4AA	0.35330	0.47410	0.15910	0.0450*	0.480 (17)
H5AA	0.45880	0.41570	0.39130	0.0490*	0.480 (17)
H6AA	0.41250	0.36990	0.63410	0.0400*	0.480 (17)

### Atomic displacement parameters (Å^2^)

**Table:**

	*U*^11^	*U*^22^	*U*^33^	*U*^12^	*U*^13^	*U*^23^
O1	0.0234 (4)	0.0505 (6)	0.0224 (4)	0.0004 (3)	0.0069 (3)	0.0096 (3)
N1	0.0334 (5)	0.0409 (6)	0.0276 (5)	−0.0035 (4)	0.0113 (4)	0.0100 (4)
N2	0.0239 (4)	0.0438 (6)	0.0243 (5)	−0.0019 (4)	0.0069 (4)	0.0107 (4)
N3	0.0224 (5)	0.0857 (10)	0.0292 (5)	−0.0024 (5)	0.0086 (4)	0.0155 (5)
C1B	0.045 (2)	0.0227 (15)	0.0208 (13)	0.0012 (14)	0.0088 (15)	0.0032 (10)
C2B	0.045 (2)	0.0379 (17)	0.0259 (14)	0.0056 (16)	0.0156 (14)	0.0043 (11)
C3B	0.059 (3)	0.0386 (18)	0.0276 (14)	0.0065 (18)	0.0197 (17)	0.0131 (11)
C4B	0.065 (3)	0.0320 (15)	0.0343 (18)	−0.0086 (16)	0.028 (2)	0.0002 (13)
C5B	0.055 (3)	0.0457 (19)	0.050 (2)	−0.0018 (19)	0.031 (2)	0.0083 (17)
C6B	0.046 (2)	0.0458 (19)	0.041 (2)	0.0010 (18)	0.0182 (19)	0.0113 (17)
C7	0.0638 (9)	0.0303 (6)	0.0298 (6)	0.0083 (6)	0.0238 (6)	0.0061 (5)
C8	0.0488 (7)	0.0349 (6)	0.0335 (6)	0.0037 (5)	0.0202 (6)	0.0097 (5)
C9	0.0389 (6)	0.0323 (6)	0.0279 (5)	0.0005 (5)	0.0126 (5)	0.0065 (4)
C10	0.0397 (7)	0.0478 (8)	0.0320 (6)	0.0081 (5)	0.0135 (5)	0.0127 (5)
C11	0.0240 (5)	0.0424 (7)	0.0238 (5)	−0.0017 (4)	0.0067 (4)	0.0040 (4)
C3A	0.056 (3)	0.044 (2)	0.0397 (18)	0.0139 (18)	0.0194 (18)	0.0134 (14)
C4A	0.055 (3)	0.0314 (17)	0.0291 (15)	−0.0016 (18)	0.017 (2)	0.0062 (12)
C5A	0.045 (3)	0.0402 (19)	0.044 (2)	0.0033 (16)	0.024 (2)	0.0081 (16)
C6A	0.038 (2)	0.0309 (16)	0.0338 (19)	0.0064 (14)	0.0144 (18)	0.0112 (13)
C2A	0.053 (2)	0.0369 (19)	0.0353 (17)	0.0087 (17)	0.0212 (16)	0.0099 (13)
C1A	0.038 (2)	0.0219 (14)	0.0268 (15)	−0.0032 (14)	0.0161 (14)	−0.0043 (12)

### Geometric parameters (Å, °)

**Table:**

O1—C11	1.2472 (15)	C5A—C6A	1.389 (10)
N1—N2	1.3788 (15)	C5B—C6B	1.389 (10)
N1—C9	1.2915 (17)	C7—C8	1.3344 (18)
N2—C11	1.3618 (15)	C8—C9	1.4610 (19)
N3—C11	1.3398 (17)	C9—C10	1.494 (2)
N2—H2B	0.8800	C2A—H2AA	0.9500
N3—H3B	0.8800	C2B—H2BA	0.9500
N3—H3C	0.8800	C3A—H3AA	0.9500
C1A—C7	1.432 (5)	C3B—H3BA	0.9500
C1A—C6A	1.391 (10)	C4A—H4AA	0.9500
C1A—C2A	1.390 (7)	C4B—H4BA	0.9500
C1B—C2B	1.390 (7)	C5A—H5AA	0.9500
C1B—C6B	1.390 (11)	C5B—H5BA	0.9500
C1B—C7	1.556 (6)	C6A—H6AA	0.9500
C2A—C3A	1.389 (8)	C6B—H6BA	0.9500
C2B—C3B	1.390 (9)	C7—H7A	0.9500
C3A—C4A	1.391 (12)	C8—H8A	0.9500
C3B—C4B	1.390 (13)	C10—H10B	0.9800
C4A—C5A	1.391 (9)	C10—H10C	0.9800
C4B—C5B	1.391 (9)	C10—H10A	0.9800
			
N2—N1—C9	118.31 (11)	C3A—C2A—H2AA	120.00
N1—N2—C11	118.53 (11)	C1A—C2A—H2AA	120.00
N1—N2—H2B	121.00	C3B—C2B—H2BA	120.00
C11—N2—H2B	121.00	C1B—C2B—H2BA	120.00
C11—N3—H3B	120.00	C4A—C3A—H3AA	120.00
H3B—N3—H3C	120.00	C2A—C3A—H3AA	120.00
C11—N3—H3C	120.00	C4B—C3B—H3BA	120.00
C6A—C1A—C7	117.1 (4)	C2B—C3B—H3BA	120.00
C2A—C1A—C7	122.6 (5)	C5A—C4A—H4AA	120.00
C2A—C1A—C6A	119.9 (5)	C3A—C4A—H4AA	120.00
C2B—C1B—C7	113.5 (6)	C5B—C4B—H4BA	120.00
C2B—C1B—C6B	120.0 (6)	C3B—C4B—H4BA	120.00
C6B—C1B—C7	126.4 (5)	C4A—C5A—H5AA	120.00
C1A—C2A—C3A	120.1 (6)	C6A—C5A—H5AA	120.00
C1B—C2B—C3B	120.0 (7)	C4B—C5B—H5BA	120.00
C2A—C3A—C4A	120.0 (5)	C6B—C5B—H5BA	120.00
C2B—C3B—C4B	120.0 (5)	C5A—C6A—H6AA	120.00
C3A—C4A—C5A	120.0 (6)	C1A—C6A—H6AA	120.00
C3B—C4B—C5B	120.0 (6)	C5B—C6B—H6BA	120.00
C4A—C5A—C6A	120.0 (8)	C1B—C6B—H6BA	120.00
C4B—C5B—C6B	120.0 (8)	C1A—C7—H7A	114.00
C1A—C6A—C5A	120.0 (6)	C1B—C7—H7A	126.00
C1B—C6B—C5B	120.0 (6)	C8—C7—H7A	114.00
C1B—C7—C8	119.7 (3)	C9—C8—H8A	117.00
C1A—C7—C8	132.9 (3)	C7—C8—H8A	117.00
C7—C8—C9	126.08 (15)	C9—C10—H10C	110.00
N1—C9—C8	114.27 (13)	C9—C10—H10B	109.00
C8—C9—C10	120.69 (11)	H10B—C10—H10C	109.00
N1—C9—C10	125.04 (12)	H10A—C10—H10B	110.00
N2—C11—N3	117.53 (11)	H10A—C10—H10C	109.00
O1—C11—N3	122.23 (11)	C9—C10—H10A	110.00
O1—C11—N2	120.24 (12)		
			
C9—N1—N2—C11	−173.34 (11)	C2B—C1B—C7—C8	−178.1 (3)
N2—N1—C9—C8	−179.53 (10)	C6B—C1B—C7—C8	2.8 (6)
N2—N1—C9—C10	1.37 (19)	C1B—C2B—C3B—C4B	0.0 (7)
N1—N2—C11—O1	179.22 (11)	C2B—C3B—C4B—C5B	0.0 (7)
N1—N2—C11—N3	−1.40 (17)	C3B—C4B—C5B—C6B	0.0 (8)
C6B—C1B—C2B—C3B	0.0 (7)	C4B—C5B—C6B—C1B	0.0 (8)
C7—C1B—C2B—C3B	−179.2 (4)	C1B—C7—C8—C9	175.5 (2)
C2B—C1B—C6B—C5B	0.0 (8)	C7—C8—C9—N1	178.13 (13)
C7—C1B—C6B—C5B	179.1 (4)	C7—C8—C9—C10	−2.7 (2)

### Hydrogen-bond geometry (Å, °)

**Table:**

Cg1 and Cg2 are the centroids of the disordered benzene rings C1A –C6A and C1B–C6B, respectively.

**Table:**

*D*—H···*A*	*D*—H	H···*A*	*D*···*A*	*D*—H···*A*
N2—H2B···O1^i^	0.88	2.12	2.9785 (15)	166
N3—H3B···O1^ii^	0.88	2.08	2.9434 (14)	168
N3—H3C···N1	0.88	2.28	2.6397 (16)	104
C10—H10A···O1^i^	0.98	2.51	3.2384 (17)	131
C10—H10B···N1^iii^	0.98	2.58	3.4566 (19)	148
C4B—H4BA···Cg1^iv^	0.95	2.86	3.618 (5)	138
C4A—H4AA···Cg1^iv^	0.95	2.76	3.590 (5)	146
C4A—H4AA···Cg2^iv^	0.95	2.93	3.714 (5)	141

Symmetry codes: (i) −*x*+1/2, −*y*+1/2, −*z*+3; (ii) −*x*+1, *y*, −*z*+7/2; (iii) −*x*+1/2, −*y*+1/2, −*z*+2; (iv) *x*, −*y*+1, *z*−1/2.
